# Durable Response of Spinal Chordoma to Combined Inhibition of IGF-1R and EGFR

**DOI:** 10.3389/fonc.2016.00098

**Published:** 2016-05-02

**Authors:** Tamara Aleksic, Lisa Browning, Martha Woodward, Rachel Phillips, Suzanne Page, Shirley Henderson, Nicholas Athanasou, Olaf Ansorge, Duncan Whitwell, Sarah Pratap, A. Bassim Hassan, Mark R. Middleton, Valentine M. Macaulay

**Affiliations:** ^1^Department of Oncology, Old Road Campus Research Building, Oxford, UK; ^2^Department of Cellular Pathology, NIHR Oxford Biomedical Research Centre, John Radcliffe Hospital, Oxford University Hospitals NHS Foundation Trust, Oxford, UK; ^3^Oxford Cancer and Haematology Centre, Churchill Hospital, Oxford University Hospitals NHS Foundation Trust, Oxford, UK; ^4^Department of Radiology, Oxford Cancer and Haematology Centre, Churchill Hospital, Oxford University Hospitals NHS Foundation Trust, Oxford, UK; ^5^BRC Oxford Molecular Diagnostic Centre, John Radcliffe Hospital, Oxford University Hospitals NHS Foundation Trust, Oxford, UK; ^6^Nuffield Department of Orthopaedics, Rheumatology and Musculoskeletal Science, Nuffield Orthopaedic Centre, Oxford, UK; ^7^Nuffield Department of Clinical Neurosciences, John Radcliffe Hospital, Oxford, UK

**Keywords:** chordoma, IGF-1R, EGFR, tyrosine kinase inhibitor, nuclear IGF-1R

## Abstract

Chordomas are rare primary malignant bone tumors arising from embryonal notochord remnants of the axial skeleton. Chordomas commonly recur following surgery and radiotherapy, and there is no effective systemic therapy. Previous studies implicated receptor tyrosine kinases, including epidermal growth factor receptor (EGFR) and type 1 insulin-like growth factor receptor (IGF-1R), in chordoma biology. We report an adult female patient who presented in 2003 with spinal chordoma, treated with surgery and radiotherapy. She underwent further surgery for recurrent chordoma in 2008, with subsequent progression in pelvic deposits. In June 2009, she was recruited onto the Phase I OSI-906-103 trial of EGFR inhibitor erlotinib with linsitinib, a novel inhibitor of IGF-1R/insulin receptor (INSR). Treatment with 100 mg QD erlotinib and 50 mg QD linsitinib was well-tolerated, and after 18 months a partial response was achieved by RECIST criteria. From 43 months, a protocol modification allowed intra-patient linsitinib dose escalation to 50 mg BID. The patient remained stable on trial treatment for a total of 5 years, discontinuing treatment in August 2014. She subsequently experienced further disease progression for which she underwent pelvic surgery in April 2015. Analysis of DNA extracted from 2008 (pre-trial) tissue showed that the tumor harbored wild-type EGFR, and a PIK3CA mutation was detected in plasma, but not tumor DNA. The 2015 (post-trial) tumor harbored a mutation of uncertain significance in ATM, with no detectable mutations in other components of a 50 gene panel, including EGFR, PIK3CA, and TP53. By immunohistochemistry, the tumor was positive for brachyury, the molecular hallmark of chordoma, and showed weak–moderate membrane and cytoplasmic EGFR. IGF-1R was detected in the plasma membrane and cytoplasm and was expressed more strongly in recurrent tumor than the primary. We also noted heterogeneous nuclear IGF-1R, which has been linked with sensitivity to IGF-1R inhibition. Similar variation in IGF-1R expression and subcellular localization was noted in 15 further cases of chordoma. In summary, this exceptionally durable response suggests that there may be merit in evaluating combined IGF-1R/INSR and EGFR inhibition in patients with chordomas that recur following failure of local treatment.

## Introduction

Chordomas are rare primary malignant bone tumors arising from primitive notochord remnants, and account for ~17% of primary malignant bone tumors of the axial skeleton (1). Chordomas express the transcription factor brachyury (T) that directs notochord development (2). Germ-line duplication of the brachyury gene is associated with familial chordoma, copy number gain is reported in sporadic chordomas, and common genetic variants in brachyury have recently been associated with both familial and sporadic chordomas (3–6).

Clinically, there is approximately equal distribution between cranial, spinal, and sacral origin, with risk of local recurrence in ~45%, metastasis in ~30%, and median survival of 6.3 years (7–9). The mainstay of treatment is local therapy in the form of surgery and radiotherapy, ideally with proton beam therapy (9). Invasion of adjacent muscles is common, compromising the likelihood of complete surgical resection, and extent of resection, surgical margins, tumor grade and proton therapy are the most important predictors of recurrence and survival (10–12). Although chordomas are typically indolent, recurrent tumors can pursue a more aggressive course with distant metastases. In a series of 18 sacral chordoma patients, 12 patients experienced recurrence, and of those, 7 died in median 5.4 months (range 4–40 months) after the recurrence (10). Chordomas are resistant to chemotherapy, and there are no licensed options for systemic therapy, although several targeted agents have induced responses in individual cases (13).

## Description of Case

**Figure 1:**
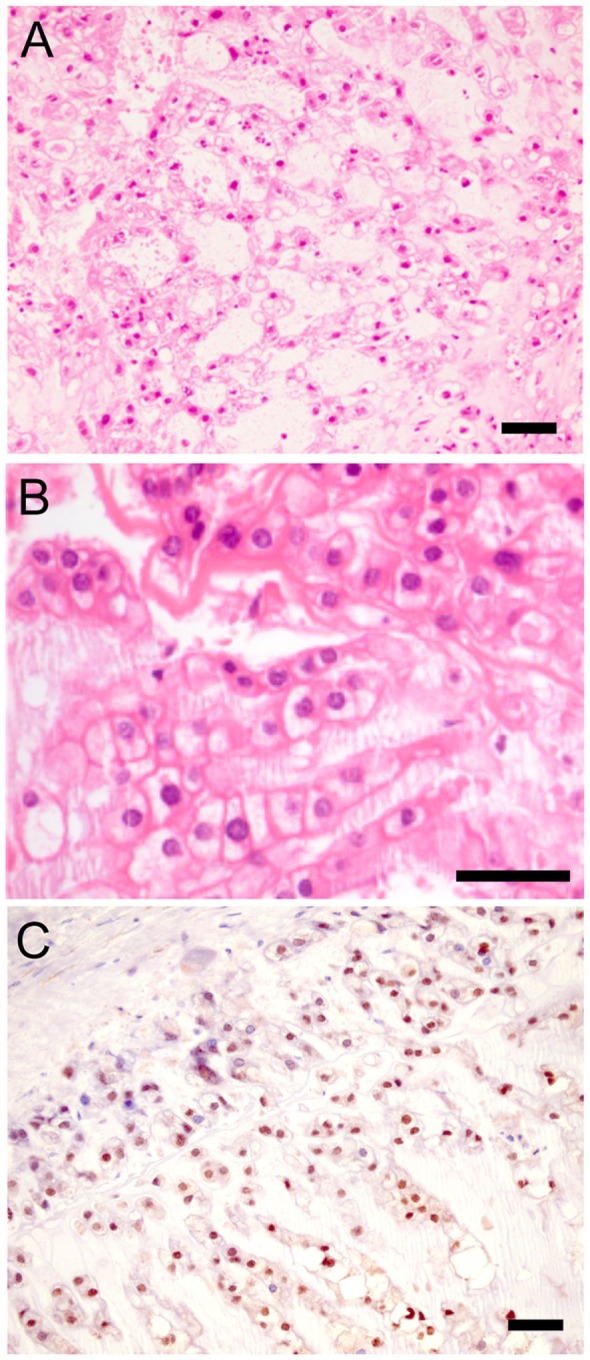
**Histological appearance of chordoma**. **(A,B)** Sections of primary tumor stained with hematoxylin and eosin (H&E). **(C)** Parallel section stained for brachyury. Scale bar: 50 μm.

The patient is a 65-year-old non-smoker who presented in mid-2003 with a 9-month history of lower back pain. Magnetic resonance imaging (MRI) revealed replacement of the L4 vertebra by a lobulated tumor that invaded the spinal canal, encroaching on the cauda equina. MRI and computed tomography (CT) scans showed no evidence of primary tumor elsewhere. She underwent biopsy under fluoroscopic control followed by surgery on 16th July 2003. The procedure involved radical tumor resection with insertion of an intervertebral cage containing a biphasic calcium phosphate ceramic (Triosite). Histological examination (Figures 1A,B) showed tumor composed predominantly of physaliphorous cells arranged in nests and set in a mucinous matrix. There was considerable nuclear pleomorphism and hyperchromasia and occasional atypical mitoses. Tumor cells were positive for cytokeratins and S100 (not shown), and also for brachyury (Figure 1C), the molecular hallmark of chordoma (2). The tumor infiltrated soft tissue and extended to the surgical margins. Proton therapy being unavailable (14), she underwent a course of adjuvant photon beam radiotherapy to the lumbar spine (L3–L5 inclusive, 50 Gy total dose in 30 fractions).

From April 2007, the patient experienced recurrent pain, weakness, and altered sensation in the lower limbs. Initial MRI scans were equivocal for tumor recurrence but, in August 2007, showed definite evidence of recurrence to the left of the fourth lumbar vertebral body, eroding the superior end-plate and the posterosuperior border of L5 vertebral body. She experienced further clinical and radiological progression, including an acute episode of paraplegia in December 2007, and in February 2008, she underwent major revision surgery. Laparotomy revealed considerable recurrent tumor behind the body of L4 with bleeding into the spinal canal at this level, and tumor infiltrating the psoas muscles, also with hemorrhagic change. She underwent a debulking procedure with anterior vertebrectomy and anterior and posterior fusion, leaving residual tumor in the psoas muscles. Histopathological analysis confirmed recurrent brachyury-positive chordoma in the L4 vertebral body, epidural surface, psoas muscles bilaterally, and left abdominal wall. Post-operatively, there was gradual recovery of power in the lower limbs, although with persistent altered sensation in the legs and loss of knee and ankle reflexes. MRI scans in March 2009 showed further evidence of progression, prompting referral to our Early Phase Clinical Trials Unit in June 2009.

**Figure 2:**
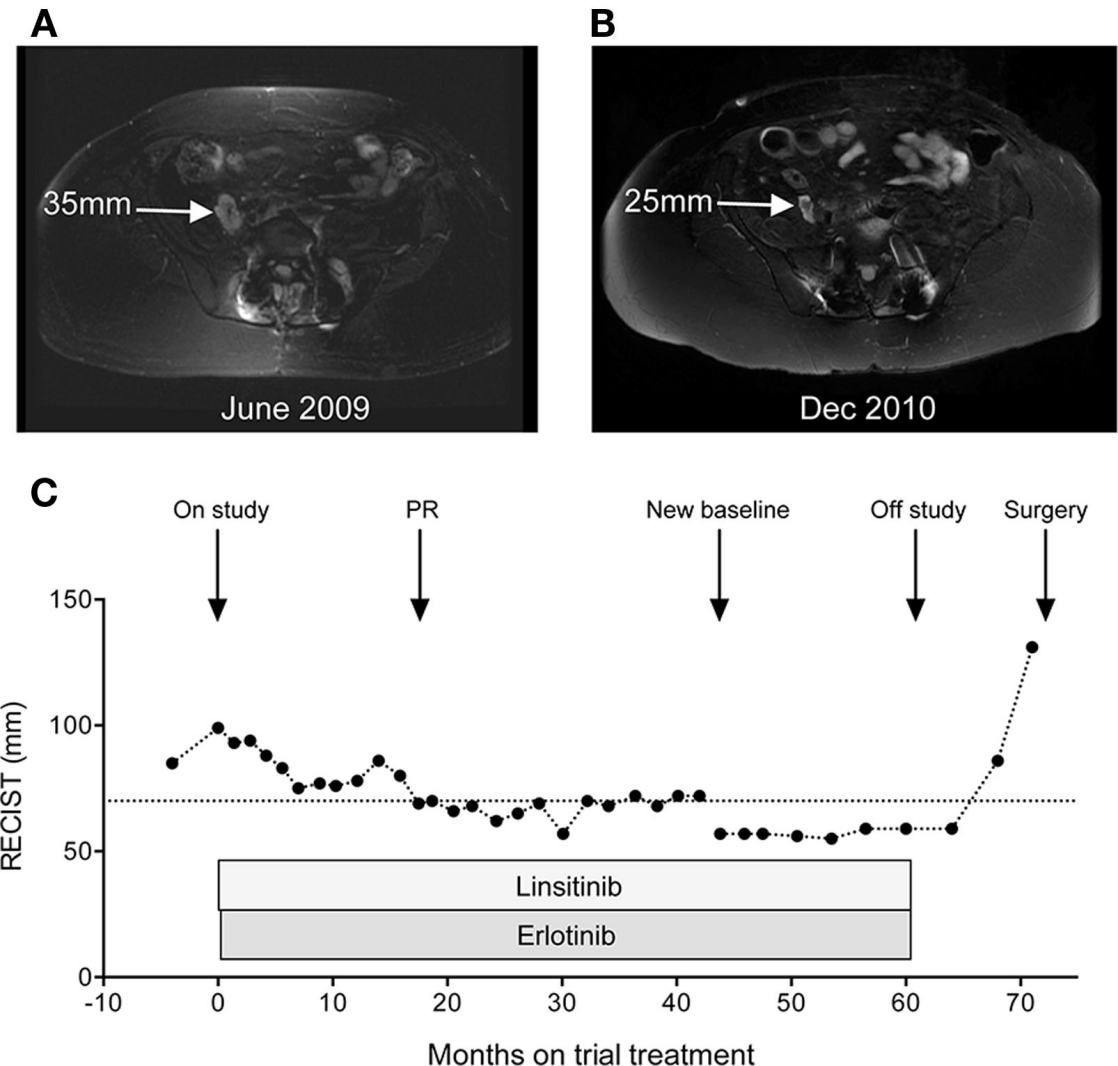
**Objective response of chordoma to linsitinib and erlotinib**. **(A)** Baseline MRI pelvis in June 2009, showing right iliopsoas deposit. Additional marker lesions were measured in the oblique right psoas (29 mm) and left paravertebral region (35 mm) giving a sum for the three lesions of 99 mm. **(B)** Response MRI of December 2010 showing reduction in size of right iliopsoas deposit. **(C)** Graph: monitoring of serial MRI scans by RECIST criteria, represented as the sum of measurements of the three marker lesions described in **(A)**. After 44 months (February 2013), these three lesions were no longer clearly measurable, and a new marker lesion (left external iliac, 57 mm) was selected to monitor response to linsitinib dose escalation from 50 mg QD to BID.

The patient was recruited onto the Phase I OSI-906-103 trial, evaluating linsitinib (OSI-906), a novel inhibitor of the type 1 insulin-like growth factor receptor (IGF-1R) and insulin receptor (INSR), in combination with the epidermal growth factor receptor (EGFR) inhibitor erlotinib (15). Initial investigations showed that hematology, renal and liver function, coagulation screen, fasting glucose, and HbA1c were all normal, with LDH 232 U/L (normal 100–190 U/L). Baseline CT scan in June 2009 showed extensive bone destruction and surgical fixation involving the lumbosacral spine. MRI, on the same date, showed recurrent tumor with measurable components within the right iliopsoas muscle (mid-right psoas 35 mm, Figure 2A; right side of L3/4, 29 mm) and to the left side of the intervertebral device at L4 (35 mm). The patient was treated on the S2 (daily linsitinib) schedule (15), receiving 50 mg linsitinib QD from 30th June 2009, with addition of 100 mg erlotinib QD after 1 week. The treatment was well-tolerated aside from dry skin (grade 1), abnormal hair and eyelash growth (grade 1), and bilateral hallux paronychia (grade 2 at worst), attributed to erlotinib, and diarrhea (grade 1), attributed to both agents. There was no evidence of hematologic toxicity, hyperglycemia, or hyperinsulinemia, and no biochemical abnormalities with the exception of creatine kinase, which was measured for the first time in March 2012 and was elevated (333 U/L, normal range 24–195 U/L; grade 1 elevation), with subsequent persistent elevation (350–520, grade 2) probably attributable to psoas muscle involvement.

Sequential MRI scans revealed initial disease stabilization, with gradual improvement to partial response by response evaluation criteria in solid tumors (RECIST) in December 2010, after 18 months of study treatment (Figures 2B,C). In February 2013, following protocol amendment to allow intra-patient dose escalation, the linsitinib dose was increased to 50 mg BID. This treatment was monitored on a new marker lesion (left external iliac deposit, 57 mm), since the previously monitored marker lesions were no longer evaluable. The patient continued on linsitinib 50 mg BID and erlotinib 100 mg QD with stabilization of the left external iliac deposit (Figure 2C). From early 2014, she experienced gradual worsening of right leg weakness. Although there was no major change in the dimensions of the monitored marker lesion, there was evidence of slow progression in non-marker lesions including deposits in the right psoas and left iliopsoas on MRI scan and left inguinal and external iliac lymphadenopathy visible on CT scan. She came off study in August 2014 after 61 months of trial treatment, and subsequently experienced further symptomatic deterioration accompanied by progression in the left external iliac deposit (Figure 2C), prompting further pelvic surgery in April 2015.

Analysis of tumor tissue was conducted under study 07/H0606/120, approved by National Research Ethics Service (NRES) Committee South Central-Oxford C. Formalin-fixed, paraffin-embedded (FFPE) tumor blocks from the 2008 surgery yielded a small amount of genomic DNA for mutation analysis. No variants were detected in EGFR codons 18, 19, and 21 (with technical failure of the exon 20 assay), KRAS, BRAF, or PIK3CA, whereas analysis of plasma DNA revealed E542K mutation in exon 9 of PIK3CA, as reported in Ref. (15). DNA was also extracted from macro-dissected FFPE tumor from the 2015 resection and was tested for mutation hotspots by targeted massively parallel sequencing (http://www.oxford-translational-molecular-diagnostics.org.uk/). This analysis showed no evidence of mutation in EGFR, TP53, PTEN, PIK3CA, PDGFRA, KRAS, NRAS, BRAF, KIT, ERBB4, FGFR1, ERBB2, MET, FGFR3, FLT3, GNAS, SMARCB1, CTNNB1, CDKN2A, ABL1, NOTCH1, PTPN11, SMO, SMAD4, VHL, NPM1, MPL, CSF1R, HRAS, JAK3, AKT1, IDH1, CDH1, FGFR2, SRC, KDR, ALK, JAK2, MLH1, RB1, HNF1A, APC, RET, STK11, FBXW7, EZH2, GNA11, GNAQ, and IDH2. A mutation of uncertain clinical significance was detected in the ATM gene [c.9031A>G, p.(M3011V), NM_000051.3].

**Figure 3:**
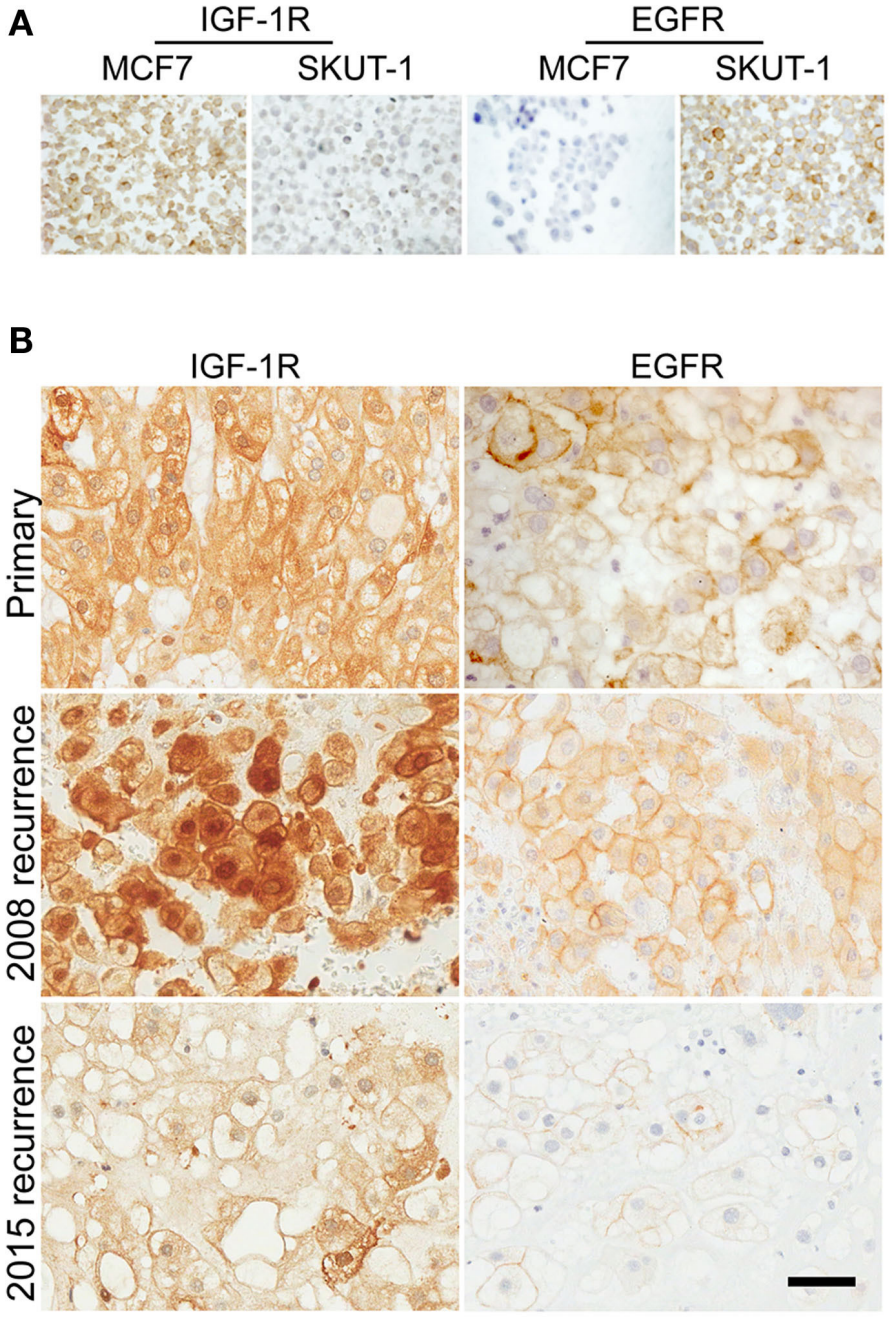
**Chordoma of trial patient contains prominent nuclear IGF-1R and membrane EGFR**. **(A)** The specificity of IGF-1R and EGFR IHC was tested using sections of formalin-fixed, paraffin-embedded MCF7 breast cancer cells (high IGF-1R, low EGFR) and SKUT-1 leiomyosarcoma cells (IGF-1R deficient, EGFR positive). **(B)** Sections of chordoma from the trial patient were stained for IGF-1R and EGFR, including primary tumor from 2003, and recurrent tumor from 2008 (pre-trial) and 2015 (post-trial). Scale bar: 50 μm.

Immunohistochemical (IHC) analysis was conducted on FFPE tumor to investigate IGF-1R and EGFR expression, using an IGF-1R IHC protocol recently optimized for sensitivity and specificity (16–18). Initial testing in FFPE control cell line pellets confirmed the specificity of the IGF-1R and EGFR antibodies in IHC (Figure 3A). Both the primary and recurrent chordoma of the trial patient contained patchy IGF-1R signal that was weaker in the primary biopsy and one of the blocks (C, psoas deposit) of recurrent tumor and stronger and more uniformly positive in two further blocks (A, B; deposits in connective tissue) from the 2008 (pre-trial) recurrence (Figure 3B). In all tumor samples, there was detectable membrane and cytoplasmic IGF-1R, with clearly apparent nuclear positivity that showed heterogeneity between different regions of tumor, and was most evident in the 2008 (pre-trial) recurrence. The tumor also contained detectable EGFR, with weak/moderate membrane and cytoplasmic signal but no nuclear positivity (Figure 3B). Analysis of tumor from the 2015 (post-trial) recurrence showed that EGFR and IGF-1R were still detectable, but with reduced intensity compared with the 2008 recurrence, and reduced nuclear IGF-1R (Figure 3B).

**Figure 4:**
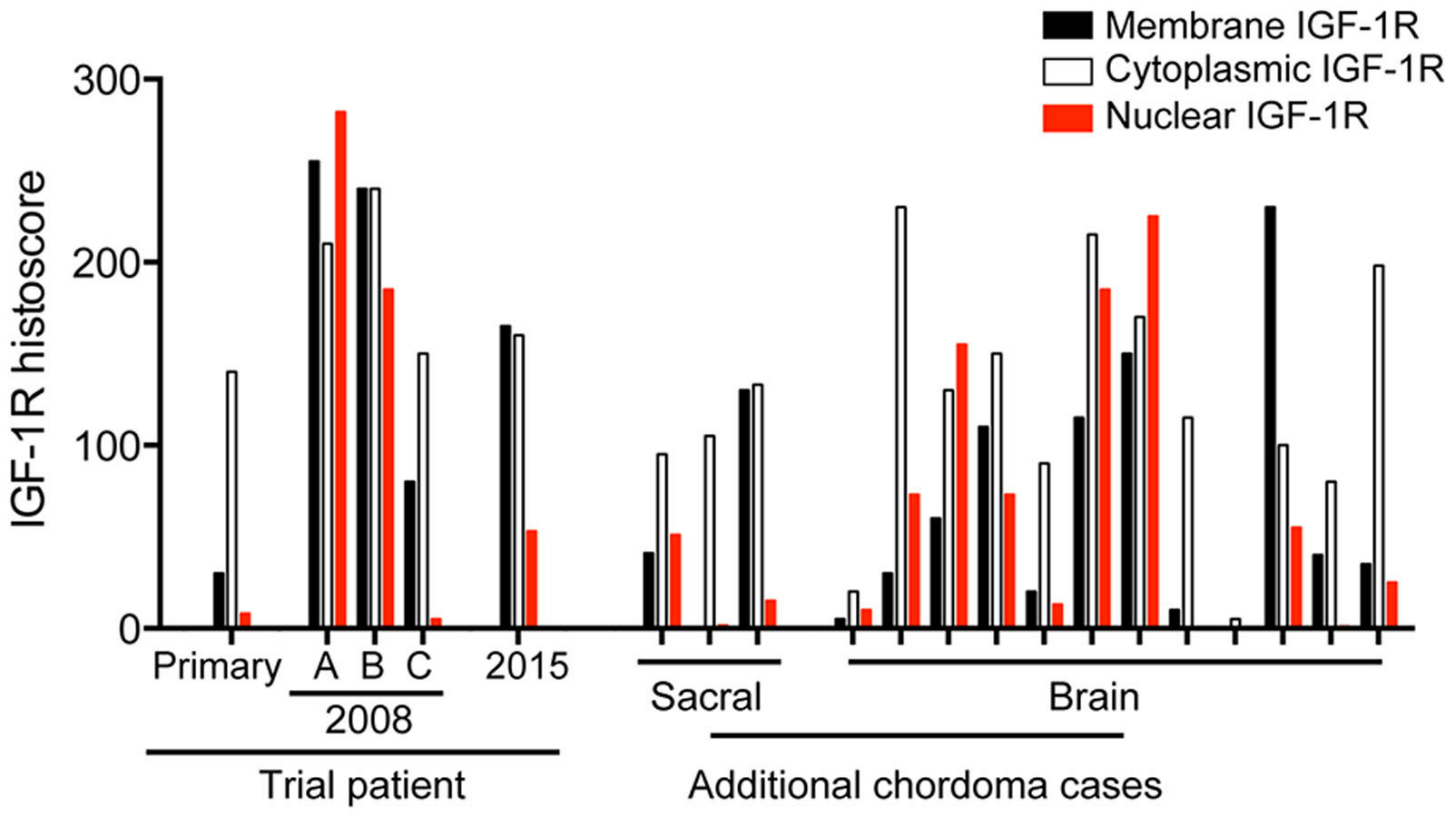
**Brain and spinal chordomas contain IGF-1R with variable membrane, cytoplasmic, and nuclear positivity**. IGF-1R IHC was performed on the primary tumor, 3 tumor blocks from the 2008 recurrence and one block from the 2015 (post-trial) recurrence of the trial patient, and 15 additional chordoma cases. Each tumor was scored for IGF-1R signal in the membrane, cytoplasm, and nucleus for the percentage showing zero (0), weak (1), moderate (2), or heavy (3) IGF-1R intensity, giving a maximum histoscore in each subcellular location of 100% × 3 = 300.

To determine the extent to which these appearances are representative, we performed IGF-1R IHC on 15 additional chordoma cases (Figure 4), including 3 cases originating in the sacrococcygeal region and 12 cases arising in the brain. All were positive for brachyury (not shown), with heterogeneity of IGF-1R expression and subcellular localization, similar to that seen in our trial case. We scored IGF-1R in the membrane, cytoplasm, and nucleus, assessing the percentage of each sample showing zero (0), weak (1), moderate (2), or heavy (3) IGF-1R intensity. Figure 4 shows the sum of IGF-1R scores in each subcellular location, indicating that the variation in IGF-1R expression and localization in our trial case had parallels in other chordoma cases.

## Discussion

Previous reports have implicated receptor tyrosine kinase (RTK) signaling in the biology of chordomas (19). A recent study reported that most chordomas express multiple RTKs, including PDGFR isoforms, EGFR, HER2, c-MET, and KIT. Like IGF-1R, all of these RTKs signal *via* AKT to enhance tumor cell growth and survival (20), and the level of AKT phosphorylation was found to correlate with poor survival (21). In several series, which have been of limited size due to the rarity of this tumor type, imatinib-induced blockade of PDGFR and KIT has shown modest activity as monotherapy (14, 22–24), with evidence of activity in combination with rapamycin (25). EGFR expression has been detected by IHC in ~70% of chordomas, with high level EGFR copy number gain in 40%, and suppression of *in vitro* chordoma cell growth by a tyrphostin EGFR inhibitor (26). As in other reported cases of chordoma, our patient’s tumor lacks the EGFR mutations that are associated with response to EGFR inhibition in lung cancer (26, 27). A recent Phase II trial of lapatinib reported stable disease as best response (28), but there are four clinical case reports of objective responses to EGFR inhibition using erlotinib, gefitinib, and/or cetuximab (29–32). Our patient was taking 100 mg erlotinib daily, below the recommended dose of 150 mg, although there is reported overlap in drug exposure in patients treated at 100 or 150 mg erlotinib daily (33).

Recent studies have investigated the contribution of the IGF axis to chordoma biology. IGF-binding protein-2 (IGFBP-2) was among the genes found to be upregulated in chordoma compared with benign intervertebral disk (34). In other cellular models and tumor types IGFBP-2 has been shown to promote tumor growth and metastasis (35). Activation of IGF-1R and INSR is reportedly detectable in ~40% of chordomas and is associated with significant reduction in disease-free survival (36). Another IHC study detected IGF-1R, IGF-1, and IGF-2 in 76, 90, and 50% of chordomas, respectively, with one-third showing moderate to strong IGF-1R, as in our study, and a significant correlation between IGF-1R staining intensity and primary tumor volume (37). This study detected membrane and cytoplasmic IGF-1R but no nuclear signal (37), using an antibody to the alpha subunit of IGF-1R, which we have found is capable of detecting nuclear IGF-1R but generally gives stronger membrane signal (16). Prominent IGF-1R nuclear positivity in the tumor of our patient is of interest, given our previous finding that nuclear IGF-1R in prostate cancer models is induced by ligand and blocked by IGF-1R kinase inhibition (16). This raises the question as to whether the presence of nuclear IGF-1R indicates strong IGF axis activation, perhaps to the point where the tumor is dependent on IGF signaling. Supporting this concept, the tumors of sarcoma patients who derived benefit from therapeutic IGF-1R antibody were reported to show exclusively nuclear IGF-1R (38).

Genetic analysis of human chordomas has identified several additional changes that are implicated in tumorigenesis and could be relevant to response to therapy. First, PIK3CA (E545K) was detected in 1 of 45 patients (39) and was also detected in the circulating DNA of our patient, although its significance is unclear, since the same mutation was not detected in the tumor. PIK3CA mutation might be predicted to mediate resistance to inhibition of signaling at the RTK level, and indeed experimental and clinical data suggest this is the case for EGFR inhibition (40, 41). The same may not be true for IGF signaling: it is known that PIK3CA-mutated breast cancer cells remain responsive to IGF stimulation and are growth delayed by IGF-1R inhibition (42–44). Second, studies of genetic alterations in chordoma identified mutation or loss of the cell cycle regulator CDKN2A (39, 45), which is of interest since we identified CDKN2C and CDKN3 as hits in a screen for proteins whose depletion enhances sensitivity to IGF-1R inhibition (46). Finally, our patient’s tumor harbored wild-type TP53, reported by our group and others to be associated with response to small molecule IGF-1R inhibitors including linsitinib in preclinical studies (47, 48).

In conclusion, we report a durable objective response to linsitinib and erlotinib in a patient with spinal chordoma that had recurred following failure of local treatment. Factors that may be relevant to the response include TP53, EGFR and PIK3CA status and the presence of nuclear IGF-1R, which may signify dependence on IGF-1R and sensitivity to IGF axis inhibition. We suggest that trials incorporating assessment of these parameters should evaluate the effects of combined IGF-1R/INSR and EGFR inhibition in patients with recurrent chordomas.

## Author Contributions

VM, MW, ABH, SP, DW, and MM were involved in care of the patient; TA, LB, RP, SP, SH, NA, and OA contributed to data acquisition and analysis; VM drafted the manuscript, and the content was reviewed, edited, and approved by all authors.

## Conflict of Interest Statement

The authors declare that the research was conducted in the absence of any commercial or financial relationships that could be construed as a potential conflict of interest.
